# Supplementary material for: Stacking Model‐Based Classifiers for Dealing With Multiple Sets of Noisy Labels
Source: Biom J. 2025 Mar 12;67(2):e70042. doi: 10.1002/bimj.70042 (PMC11898607; doi:10.1002/bimj.70042)
Supplement: Supplementary file 1 — Supporting Information [file BIMJ-67-e70042-s001.zip › density-based-ensemble-model-main/competitors_real_data/netlab-master/netlab-master/help/glmerr.htm]

Netlab Reference Manual glmerr


# glmerr

## Purpose

Evaluate error function for generalized linear model.

## Synopsis

```
e = glmerr(net, x, t)
[e, edata, eprior] = glmerr(net, x, t)
[e, edata, eprior, y, a] = glmerr(net, x, t)
```

## Description

`e = glmerr(net, x, t)` takes a generalized
linear model data structure `net` together with a matrix `x`
of input vectors and a matrix `t` of target vectors, and evaluates
the error function `e`. The choice of error function corresponds
to the output unit activation function. Each row of `x`
corresponds to one input vector and each row of `t` corresponds to
one target vector.

`[e, edata, eprior, y, a] = glmerr(net, x, t)` also returns
the data and prior components of the total error.

`[e, edata, eprior, y, a] = glmerr(net, x)` also returns a matrix `y`
giving the outputs of the models and a matrix `a`
giving the summed inputs to each output unit, where each row
corresponds to one pattern.

## See Also

`glm`, `glmpak`, `glmunpak`, `glmfwd`, `glmgrad`, `glmtrain`

---

**Pages:**
Index


---

Copyright (c) Ian T Nabney (1996-9)
